# ERK5 Signaling and Ferroptosis in Bone Homeostasis: Emerging Connections and Therapeutic Perspectives

**DOI:** 10.3390/biom16101521

**Published:** 2026-10-19

**Authors:** Jiangying Zhang, Xiping Dou, Haochuan You, Xiuyuan Wang, Yixiang Zhao, Bin Geng, Yayi Xia

**Affiliations:** 1 Department of Orthopedics, Lanzhou University Second Hospital, Lanzhou 730030, China; zhjiangying2025@lzu.edu.cn (J.Z.); douxp20@lzu.edu.cn (X.D.); youhch2024@lzu.edu.cn (H.Y.); wxiuyuan2024@lzu.edu.cn (X.W.); zhyixiang2024@lzu.edu.cn (Y.Z.);; 2 Intelligent Orthopedic Industry Technology Center of Gansu Province, Orthopedic Clinical Medical Research Center, Lanzhou 730030, China; 3 The Second School of Clinical Medicine, Lanzhou University, Lanzhou 730030, China

**Keywords:** ERK5, MAPK7, ferroptosis, bone homeostasis, oxidative stress, bone remodeling, osteoporosis, regulated cell death

## Abstract

Bone homeostasis depends on coordinated activity among bone marrow mesenchymal stem cells, osteoblasts, osteocytes, and osteoclasts. Disruption of this cellular network contributes to osteoporosis, impaired fracture repair, and other skeletal disorders, while redox stress and regulated cell-death programs increasingly appear to shape the behavior of these skeletal populations. Ferroptosis is an iron-dependent form of regulated cell death driven by phospholipid peroxidation and failure of antioxidant defenses. Preclinical studies indicate that ferroptotic injury can compromise osteogenic differentiation, osteoblast function, and osteocyte integrity; in osteoclasts, the biological effect is more model-dependent. Extracellular signal-regulated kinase 5 (ERK5/MAPK7) integrates mechanical, oxidative, inflammatory, and metabolic cues, but skeletal studies have reported divergent effects across genetic, pharmacological, developmental, and mechanically stimulated models. Direct evidence that ERK5 regulates ferroptosis in skeletal cells is still lacking. This review evaluates ferroptosis and ERK5 signaling as potentially interacting stress-response systems, separates established findings from indirect and hypothetical links, and examines how ERK5-dependent stress adaptation, mitochondrial regulation, and metabolic state may modify ferroptotic susceptibility. We propose that ERK5 may shift the threshold between adaptive survival and ferroptotic injury in a cell-, stress-, and disease-stage-dependent manner, providing a testable framework for skeletal degeneration and regeneration.

## 1. Introduction

Bone is a dynamic tissue that remodels throughout life through coordinated activity of osteoblasts, osteoclasts, osteocytes, and marrow stromal progenitors. This coupling maintains skeletal integrity and enables adaptation to mechanical and metabolic demand; disruption by aging, oxidative stress, inflammation, or metabolic disease contributes to bone loss and impaired regeneration [1,2,3].

Canonical regulators such as RANK/RANKL signaling and Wnt/β-catenin are central to skeletal remodeling [1,4]. Ferroptosis adds a distinct layer of regulation because it is driven by iron-dependent phospholipid peroxidation and is constrained by antioxidant systems that include the system Xc^−^/GSH/GPX4 pathway, FSP1-CoQ10, and Nrf2-associated redox programs [5,6,7,8,9].

Bone-focused work now implicates ferroptotic injury in BMSCs, osteoblasts, osteocytes, and osteoclast precursors, but the biological consequence is not uniform across these populations. In osteogenic and osteocytic compartments, ferroptosis generally compromises differentiation, function, or survival; in osteoclast precursors, it more often limits differentiation or viability. Importantly, rescue by ferroptosis-directed interventions in several cell and animal models argues for a causal contribution in at least a subset of skeletal disorders rather than a purely correlative change in markers [10,11,12,13,14].

The kinase ERK5, also known as MAPK7, is distinguished from other MAPKs by its large C-terminal transcriptional activation domain and its ability to couple extracellular stimuli to nuclear responses [15,16,17]. In bone, ERK5 participates in mechanotransduction, osteogenic differentiation, skeletal development, and osteoclast regulation, but the direction of these effects varies across developmental stages, cell lineages, mechanical environments, and experimental approaches [18,19,20,21,22,23].

A direct mechanistic link between ERK5 and ferroptosis has not been demonstrated in skeletal cells. Recent non-skeletal studies nevertheless show that ERK5-related ferroptotic phenotypes are biologically plausible and methodologically complex: ERK5 can directly promote ferroptosis in microglia, whereas commonly used ERK5 inhibitors can suppress ferroptosis through ERK5-independent mechanisms [24,25]. These findings argue against a simple linear or unidirectional ERK5–ferroptosis model.

Previous reviews have separately summarized the roles of ERK5 in skeletal biology and ferroptosis in bone metabolic disease [18,26]. The present review therefore does not aim simply to combine these two subjects. Instead, its principal contribution is to critically evaluate the evidentiary basis for a proposed ERK5–ferroptosis relationship in bone. We distinguish three levels of evidence: direct causal evidence linking ERK5 to ferroptosis, which is currently confined to non-skeletal systems; skeletal evidence showing that ERK5 signaling and ferroptosis independently regulate overlapping stress-response processes; and mechanistically plausible but as yet untested points of intersection. This framework allows us to identify which observations genuinely support an ERK5–ferroptosis hypothesis, which are merely compatible with such a model, and which should not be interpreted as evidence of ERK5 dependence. On this basis, we evaluate areas of convergence in redox regulation, mitochondrial homeostasis, and cell-fate control and derive specific experimental predictions required to establish or refute a causal ERK5–ferroptosis relationship in skeletal cells.

### 1.1. Literature Search and Evidence Assessment

This article is a critical narrative rather than a systematic review. Three targeted searches were conducted in PubMed on 6 October 2026. All search terms were specified in the Title/Abstract field. The searches addressed ferroptosis-related processes in skeletal systems, MEK5–ERK5 signaling in skeletal systems, and ERK5–ferroptosis connections without a skeletal restriction, respectively. The exact Boolean queries were as follows:

#### 1.1.1. Search 1 Ferroptosis and Skeletal Biology

(ferroptosis[Title/Abstract] OR “lipid peroxidation”[Title/Abstract] OR GPX4[Title/Abstract] OR SLC7A11[Title/Abstract]) AND (bone[Title/Abstract] OR osteoblast*[Title/Abstract] OR osteoclast*[Title/Abstract] OR osteocyt*[Title/Abstract] OR BMSC*[Title/Abstract] OR “bone marrow mesenchymal stem cell*”[Title/Abstract] OR osteoporosis[Title/Abstract] OR fracture*[Title/Abstract])

#### 1.1.2. Search 2 MEK5–ERK5 Signaling and Skeletal Biology

(ERK5[Title/Abstract] OR MAPK7[Title/Abstract] OR MEK5[Title/Abstract]) AND (bone[Title/Abstract] OR osteoblast*[Title/Abstract] OR osteoclast*[Title/Abstract] OR osteocyt*[Title/Abstract] OR BMSC*[Title/Abstract] OR “mesenchymal stem cell*”[Title/Abstract] OR osteoporosis[Title/Abstract] OR fracture*[Title/Abstract])

#### 1.1.3. Search 3 ERK5 and Ferroptosis

(ERK5[Title/Abstract] OR MAPK7[Title/Abstract]) AND (ferroptosis[Title/Abstract] OR GPX4[Title/Abstract] OR SLC7A11[Title/Abstract] OR FSP1[Title/Abstract] OR DHODH[Title/Abstract] OR “lipid peroxidation”[Title/Abstract])

Reference lists of key primary studies and recent reviews were also screened for additional relevant publications.

Original experimental studies addressing ferroptosis or ERK5 signaling in skeletal cells, bone-related disease models, or mechanistically relevant non-skeletal systems were prioritized. Reviews were used primarily to provide conceptual context and to identify additional primary literature. Studies lacking sufficient mechanistic relevance to ferroptosis, ERK5 signaling, or skeletal biology were not evaluated as mechanistic support. Because this review was designed as a critical narrative synthesis rather than a systematic review or meta-analysis, no formal risk-of-bias or GRADE assessment was performed.

## 2. Ferroptosis in Bone Homeostasis

### 2.1. Molecular Mechanisms of Ferroptosis

Ferroptosis is an iron-dependent form of regulated cell death characterized by iron-dependent lipid peroxidation and mechanistically distinct from apoptosis, necroptosis, and other regulated cell-death modalities [27,28]. According to the recommendations of the Nomenclature Committee on Cell Death, regulated cell-death entities should be defined primarily by their molecular mechanisms rather than by morphology alone [29]. Since its initial description in 2012, ferroptosis has been recognized as a distinct cellular death program driven by iron accumulation, oxidative stress, and uncontrolled lipid peroxidation [5,30]. Unlike apoptosis, which is mainly mediated by caspase activation, ferroptosis is characterized by disruption of intracellular redox balance and excessive accumulation of lipid reactive oxygen species [5,6,27,28,30,31].

Iron metabolism represents a central determinant of ferroptotic sensitivity. Excess intracellular ferrous iron (Fe^2+^) promotes hydroxyl radical generation through Fenton chemistry, thereby accelerating oxidative damage to polyunsaturated fatty acid-containing membrane phospholipids. Meanwhile, impairment of antioxidant defense systems, particularly the system Xc^−^/glutathione (GSH)/glutathione peroxidase 4 (GPX4) pathway, facilitates lipid peroxide accumulation and ultimately triggers ferroptotic cell death [5,32,33,34,35].

Ferroptotic susceptibility is controlled by several partially parallel defense systems rather than by GPX4 alone. The FSP1–CoQ10–NAD(P)H system suppresses phospholipid peroxidation independently of glutathione-dependent GPX4 activity, whereas the GTP cyclohydrolase 1 (GCH1)–tetrahydrobiopterin (BH4) pathway provides an additional GPX4-independent defense mechanism. Production of BH4 through GCH1 limits ferroptosis through radical-trapping antioxidant activity and lipid remodeling, including preservation of phospholipid species containing polyunsaturated fatty acyl chains and modulation of cellular CoQ10 pools [36].

Mitochondrial ferroptosis defense is further supported by dihydroorotate dehydrogenase (DHODH), an inner-mitochondrial-membrane enzyme that reduces ubiquinone to ubiquinol and acts in parallel with mitochondrial GPX4 to suppress mitochondrial lipid peroxidation [37]. In parallel, Nrf2 coordinates broader antioxidant and iron-homeostasis programs that can modify ferroptotic susceptibility. These pathways should be regarded as general determinants of ferroptotic threshold rather than as established bone-specific ferroptosis circuits, because direct causal evidence for the GCH1–BH4 and DHODH–CoQ systems in skeletal cells remains limited.

### 2.2. Ferroptosis and Bone Marrow Mesenchymal Stem Cells

Bone marrow mesenchymal stem cells (BMSCs) are major stromal progenitors that support adult bone formation and regeneration, although marrow stromal populations are heterogeneous and spatially organized [2,38]. Their osteogenic competence is therefore a critical determinant of skeletal maintenance and repair.

Evidence for ferroptosis in BMSCs has strengthened substantially. Studies using osteoporotic or iron-loaded BMSCs, primary human cells, and in vivo osteoporosis models show that iron accumulation, oxidative stress, and impaired ferritin or GPX4-related defense can increase ferroptotic vulnerability and reduce osteogenic differentiation [10,39,40,41].

Mechanistically, BMSC ferroptosis has been linked to impaired ferritin stability, altered SLC7A11/GSH/GPX4 defense, and iron-dependent lipid peroxidation. Rescue with ferrostatin-1, iron chelation, Nrf2-directed interventions, BMP9-mediated GPX4 restoration, or other anti-ferroptotic strategies can recover osteogenic activity in several preclinical models [10,40,41,42]. Evidence quality nevertheless varies. The CRYAB–FTH1 study reported iron/ROS changes and metabolic viability with ferrostatin-1 or FTH1 rescue [10]; these findings support functional involvement but do not by themselves establish membrane-integrity-defined death or oxidized phospholipid accumulation.

**Figure 1:**
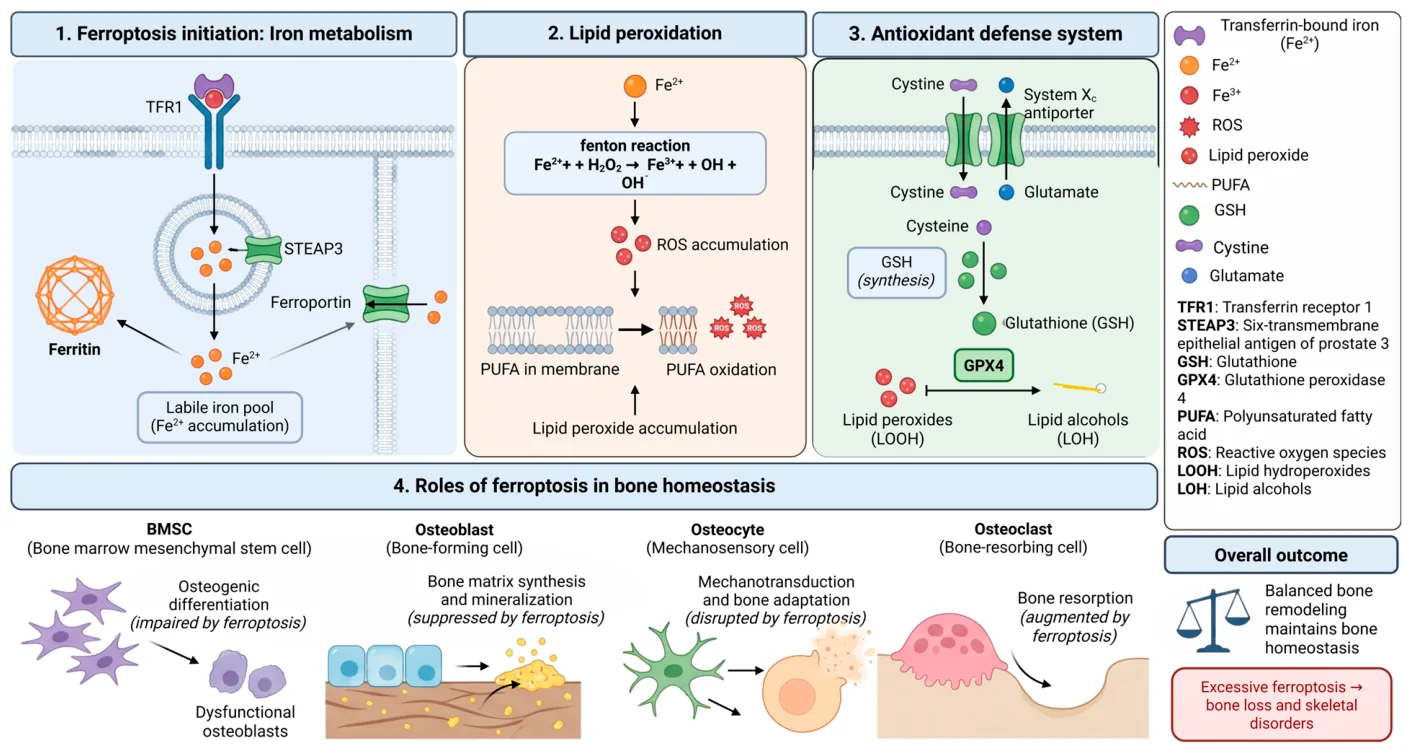
Molecular mechanisms of ferroptosis and its involvement in bone homeostasis. Transferrin receptor 1-mediated iron uptake and accumulation of redox-active Fe^2+^ promote Fenton chemistry and reactive oxygen species generation, while oxidation of polyunsaturated fatty acid-containing phospholipids drives lipid peroxide accumulation. System Xc^−^-dependent cystine uptake, glutathione synthesis, and GPX4-mediated detoxification counteract ferroptotic damage. Dysregulated ferroptosis can impair BMSC osteogenesis, osteoblast function, and osteocyte integrity. In osteoclasts, ferroptosis generally limits cell survival or differentiation, but its net effect on bone resorption depends on iron availability and the surrounding redox context. Abbreviations: STEAP3, six-transmembrane epithelial antigen of prostate 3; GSH, glutathione; GPX4, glutathione peroxidase 4; PUFA, polyunsaturated fatty acid. Created in BioRender. Zhang, J. (2026) https://BioRender.com/vdpwf0d. Agreement number: OS2AAKSAU6.

Together, these studies place ferroptosis among the mechanisms that can restrict skeletal progenitor function. A senescence model further connected Steap4-dependent Fe^2+^/ROS signaling with both ferroptosis and adipogenic differentiation of BMSCs [43]. Whether this relationship extends to lineage allocation more broadly, however, is unresolved; in many disease settings ferroptosis may still be a consequence of metabolic stress rather than the initiating determinant of cell fate. The core ferroptosis pathways and their cell-specific skeletal consequences are summarized in Figure 1.

### 2.3. Ferroptosis in Osteoblast Dysfunction

Osteoblasts synthesize bone matrix and drive mineralization, making preservation of their differentiation and survival central to bone formation [44]. Iron dysregulation and oxidative stress can compromise these functions, and several disease models now identify ferroptosis as one mechanism of osteoblast injury [11,45,46,47].

Among skeletal cell types, osteoblasts are supported by one of the more developed ferroptosis evidence bases. Iron overload, glucocorticoid exposure, hyperglycemic stress, age-associated redox imbalance, and COPB1 deficiency have each been linked to iron accumulation, lipid peroxidation, mitochondrial abnormalities, and loss of osteogenic markers. In several of these models, ferroptosis inhibition or restoration of antioxidant defense also rescues osteoblast differentiation or bone phenotypes, strengthening the case for functional involvement rather than marker association alone [11,45,46,47,48].

Antioxidant signaling related to Nrf2 is frequently protective in osteoblast models, particularly through maintenance of GPX4- and SLC7A11-associated redox defense [47,49]. However, downstream Nrf2 effectors are not uniformly anti-ferroptotic across skeletal cell types. In diabetic osteocytes, NRF2/c-JUN-driven HO-1 induction contributed to iron-dependent ferroptotic injury rather than protection [12]. This distinction argues against treating the Nrf2/HO-1 module as a universally protective ferroptosis pathway in bone.

These observations also illustrate why redox pathways should not be assigned a fixed pro- or anti-ferroptotic role without attention to cell type and disease context [12,49,50].

### 2.4. Ferroptosis in Osteocytes and Osteoclasts

Osteocytes are long-lived cells embedded within the mineralized matrix and are central mechanosensors in bone remodeling. Earlier hypoxia studies documented osteocyte stress and cell death without ferroptosis-specific validation, whereas direct in vivo and genetic studies now support osteocyte ferroptosis in diabetic, postmenopausal, aging-associated, and iron-dysregulated skeletal states [12,14,51,52,53].

The osteocyte evidence has therefore progressed beyond a purely associative stage. Genetic models involving GPX4, ATF3/TFR1-driven iron uptake, and osteocytic LCN2/SLC22A17 signaling provide mechanistic links between ferroptotic injury and altered osteoclast coupling, cortical bone loss, lacunocanalicular integrity, or osteoblast-mediated bone formation [14,52,53]. Even so, the relative contribution of ferroptosis to each skeletal disorder and its interaction with senescence and other forms of cell death remain disease-dependent.

Osteoclasts are multinucleated cells responsible for bone resorption and are essential for physiological bone remodeling. Direct studies demonstrate that osteoclast precursors can enter ferroptosis, but the relationship between iron availability, osteoclastogenesis, and ferroptotic death is more complex than in osteoblasts or osteocytes [13].

During RANKL-induced osteoclastogenesis, increased iron demand and ferritinophagy can create ferroptotic vulnerability. Hypoxia suppresses ferritinophagy and protects osteoclasts from ferroptosis [13]. Artesunate and saikosaponin A, two pleiotropic natural products, have been reported to increase lipid peroxidation, promote ferroptosis-associated cell death, and reduce osteoclast differentiation [54,55]. Their effects require interpretation alongside orthogonal death measurements, functional rescue, and genetic evidence because pharmacological exposure alone does not establish ferroptosis specificity. Conversely, NRF2 deficiency under iron overload can precipitate osteoclast ferroptosis even when iron loading promotes osteoclastogenic signaling [56].

Genetic evidence adds a further layer of complexity: MZF1 loss increases osteoclastogenesis while altering RANKL-associated Nrf2/GPX4 and ferroptosis markers [57]. Taken together, current data more strongly support ferroptosis as a brake on osteoclast survival or differentiation than as a simple driver of bone resorption. The apparent contradictions arise because iron loading and adaptive antioxidant signaling can promote osteoclastogenesis while excessive lipid peroxidation simultaneously pushes cells toward ferroptotic death [13,54,55,57].

### 2.5. Ferroptosis in Skeletal Disorders

Osteoporosis provides the most developed skeletal context in which ferroptosis has been studied. Iron dysregulation, oxidative stress, estrogen deficiency, aging, and metabolic disease can converge on ferroptosis-related mechanisms in bone-forming and regulatory cells [14,43,47,58].

The strongest disease-level evidence currently comes from osteoporosis and related models of aging, estrogen deficiency, iron excess, and metabolic stress. Across these settings, ferroptosis-associated changes have been reported in osteoblasts, osteocytes, osteoclasts, and BMSCs, and several studies combine lipid-peroxidation assays with genetic perturbation, ferroptosis-selective rescue, or in vivo skeletal phenotyping [10,13,14,58].

Aging, estrogen deficiency, iron overload, glucocorticoid exposure, and diabetic or inflammatory stress can each shift bone-cell redox balance toward ferroptotic vulnerability. In multiple models, iron chelation, ferrostatin-1, pathway-specific rescue, or restoration of GPX4/SLC7A11-related defense improves cellular or skeletal outcomes, supporting a causal contribution rather than a marker-only association in at least a subset of disease settings [11,14,40,49].

The evidence base remains predominantly preclinical. Current guidance for ferroptosis research emphasizes the need for orthogonal molecular readouts, functional rescue, and careful discrimination from other forms of cell death, and clinical efficacy of ferroptosis-directed treatment in skeletal disease has not been established [26,59].

Beyond osteoporosis, direct ferroptosis-related evidence now extends to diabetic bone disease [12,45,60], glucocorticoid-associated osteonecrosis [46,61,62], age-related cortical bone loss [52,63], and impaired fracture repair or nonunion [64,65]. The strength of evidence is not uniform: diabetic and age-related bone models include several mechanistic and in vivo studies, whereas osteonecrosis and fracture-repair data remain concentrated in a smaller number of experimental systems. No clinical trial has yet established that ferroptosis-directed therapy improves skeletal outcomes in patients. Current evidence across skeletal cell types and disease models is summarized in Table 1.

Evidence-assessment definitions: Table 1 uses descriptive evidence profiles rather than ordinal strength ratings. Marker association denotes changes in ROS, iron, GPX4/SLC7A11 abundance, or GSH; these alone do not establish ferroptotic death. Functional support requires assessment of cell death and lipid peroxidation together with concordant rescue by ferroptosis-directed interventions. Metabolic viability assays alone do not distinguish death from altered proliferation or metabolism, and rescue by radical-trapping antioxidants is not ferroptosis-exclusive. Genetic support identifies pathway perturbation or genetic rescue, which should be interpreted with death and lipid-peroxidation measurements. In vivo skeletal support denotes bone phenotyping associated with a relevant intervention; a bone phenotype alone does not establish ferroptosis as its cause. Alternative death mechanisms, intervention specificity, and consistency across models should also be assessed. The profiles summarize representative supporting experiments and remaining gaps; they do not imply that every cited study measured every listed endpoint. Cell-type and disease rows overlap and are not independent evidence sets. This is a qualitative preclinical synthesis, not a formal GRADE assessment or a measure of clinical efficacy.

## 3. Signaling Through ERK5 in Bone Homeostasis

### 3.1. Molecular Characteristics and Activation Mechanisms of ERK5 Signaling

Extracellular signal-regulated kinase 5 (ERK5), also known as mitogen-activated protein kinase 7 (MAPK7), is a unique member of the mitogen-activated protein kinase (MAPK) family. Compared with classical MAPKs, including ERK1/2, JNK, and p38, ERK5 possesses a distinctive C-terminal transcriptional activation domain in addition to its conserved N-terminal kinase domain. This structural feature enables ERK5 to function not only as a conventional kinase but also as a multifunctional signaling molecule involved in transcriptional regulation and cellular adaptation [15,16,17].

Throughout this review, gene symbols are italicized according to species-specific nomenclature: human gene symbols are written in uppercase (e.g., *MAPK7*, *GPX4*, and *SLC7A11*), mouse gene symbols are written with an initial capital letter (e.g., *Mapk7*, *Gpx4*, and *Slc7a11*; *Erk5* is retained when reflecting the terminology used in the cited study), and protein names are presented in non-italicized form (e.g., ERK5, GPX4, and SLC7A11).

Activation of ERK5 is primarily mediated by the MEK5–ERK5 signaling cascade [69,70]. Upon stimulation by extracellular factors, including growth factors, cytokines, mechanical stress, and oxidative stimuli, MEK5 phosphorylates ERK5 at specific residues within the activation loop, resulting in ERK5 kinase activation and subsequent nuclear translocation [17,71,72,73]. Activated ERK5 interacts with multiple downstream transcription factors, including members of the myocyte enhancer factor 2 (MEF2) family, thereby regulating genes involved in proliferation, survival, differentiation, and stress responses [16,69].

The dual kinase–transcriptional architecture of ERK5 makes catalytic inhibition, protein depletion, and disruption of C-terminal transcriptional activity mechanistically distinct perturbations. Several ATP-competitive inhibitors suppress ERK5 kinase activity while promoting nuclear localization and paradoxical activation of the C-terminal transcriptional activation domain (TAD) [74,75]. These effects, together with compound-specific off-target activity, complicate interpretation of skeletal inhibitor studies; the pharmacological distinctions are summarized in Section 6.2 and Table 3.

The ERK5 pathway cannot be assigned a single biological output across tissues or experimental conditions. In bone, the phenotype attributed to ERK5 varies according to the cell population and differentiation stage—for example, developmental mesenchymal progenitors, adult LepR-positive stromal cells, and differentiated osteoblast-lineage cells are biologically distinct—and according to whether cells are exposed to mechanical stimulation such as fluid shear stress or maintained under static culture conditions [19,20,21,76,77].

### 3.2. Regulation of Osteoblast Differentiation and Bone Formation by ERK5

Osteoblast studies therefore show bidirectional ERK5 effects rather than a uniform anabolic program. Differences in cell lineage, differentiation stage, mechanical stimulation, and the mode of ERK5/MEK5 manipulation are likely to account for part of the disagreement among studies [19,20,21,76].

Activity of ERK5 supports osteogenic responses in mechanically stimulated MC3T3-E1 cells and in several pro-osteogenic models, including a study of mangiferin, a plant-derived xanthone with antioxidant properties [19,20,77,78]. By contrast, MEK5 inhibition or knockdown and genetic deletion of *Erk5* in adult LepR-positive stromal cells enhanced osteogenic differentiation in other systems [21,76]. Developmental mesenchymal ERK5 is also required for normal skeletogenesis [79].

Taken together, these data indicate that developmental mesenchyme, adult stromal populations, and mechanically stimulated osteoblast-lineage cells should not be treated as interchangeable experimental compartments. Table 2 summarizes the representative models and methodological differences underlying these apparently conflicting findings [19,20,21,76,79].

### 3.3. Regulation of Osteoclast Differentiation and Bone Resorption by ERK5

Osteoclast studies report opposing effects of MEK5–ERK5 perturbation. Amano et al. found that pathway inhibition or MEK5 knockdown reduced c-Fos induction and osteoclast differentiation, consistent with a pro-osteoclastogenic role [22]. In contrast, Loveridge et al. reported increased osteoclastogenesis and bone resorption after partial genetic ERK5 loss, with a similar direction after low-dose pathway inhibition [23]. In a mechanically stimulated RAW264.7 model, fluid shear stress suppressed osteoclast differentiation through ERK5-associated signaling [80]. These findings implicate model, lineage, and loading conditions as determinants of the response.

The exposure differences are also relevant: representative experiments used approximately 4–5 μM BIX02189 and 1 μM XMD8-92 in the Amano study, compared with 100 nM BIX02188/BIX02189 in the Loveridge study [22,23]. Because these compounds have distinct target profiles, and XMD8-92 has bromodomain activity, such results are pharmacological evidence compatible with pathway involvement rather than definitive evidence of ERK5 dependence. Genetic and domain-specific comparisons are needed to determine whether the discordant phenotypes arise from ERK5 protein loss, catalytic inhibition, altered transcriptional output, or compound-specific effects. None of these osteoclast studies establishes an ERK5–ferroptosis mechanism.

### 3.4. Mechanical Adaptation of Bone Tissue Through ERK5 Signaling

Bone is mechanically responsive, and osteocytes serve as major mechanosensors that coordinate remodeling responses to loading [3]. Signaling through ERK5 is activated by fluid shear and cytoskeletal reorganization in osteoblastic and osteoclastic models, linking mechanical input to survival, proliferation, differentiation, and transcriptional responses [19,72,80,81,82].

A potentially important osteocyte-specific transcriptional link involves myocyte enhancer factor 2C (MEF2C). The kinase ERK5 can directly phosphorylate and activate MEF2C, establishing MEF2C as a well-characterized downstream transcriptional effector of ERK5 [83]. In the skeleton, MEF2C is a major regulator of *Sost* transcription through the distal evolutionarily conserved region 5 (ECR5) enhancer. Osteoblast/osteocyte-specific deletion of *Mef2c* or targeted deletion of ECR5 reduces *Sost* expression and produces a high-bone-mass phenotype associated with increased bone formation [84]. Importantly, ECR5/MEF2C-dependent control of *SOST* is stimulus-selective rather than universal; disruption of the MEF2C-binding site within ECR5 abolishes forskolin responsiveness but does not eliminate responses to 1,25-dihydroxyvitamin D3 or TGF-β1 [85]. Thus, an ERK5–MEF2C–SOST pathway represents a biologically plausible and experimentally testable link between ERK5 signaling and osteocyte-specific transcriptional control, but direct regulation of SOST by ERK5 has not yet been demonstrated in osteocytes.

Mechanical activation of ERK5 therefore provides a plausible route by which loading could alter both osteocyte transcriptional state and cellular stress tolerance. Because SOST itself is mechanically regulated, the ERK5–MEF2C–SOST relationship deserves direct testing under defined loading conditions. However, the MEF2C/ECR5 pathway should not be assumed to mediate all mechanical regulation of SOST, because ECR5-independent mechanisms of bone mechanoadaptation have also been reported [86]. Whether ERK5-dependent MEF2C activity also modifies osteocyte ferroptotic susceptibility remains unknown and should be tested independently rather than inferred from changes in SOST expression.

### 3.5. Oxidative Stress Responses Associated with ERK5 Signaling in Bone

Signaling through ERK5 also participates in general oxidative and metabolic stress responses [73,87]. Mitochondrial complex I activity can engage ERK5-dependent antioxidant programs, and ERK5 signaling has been linked to MnSOD expression in non-skeletal systems [87]. These observations support a role in general redox adaptation but should not be interpreted as direct evidence of ferroptosis-specific protection, because MnSOD primarily regulates mitochondrial superoxide rather than phospholipid hydroperoxides. In osteoblasts, ERK5 contributes to mangiferin-mediated protection against H_2_O_2_-induced oxidative injury [88], but this study did not establish ferroptosis-specific protection. Collectively, these findings support an ERK5-associated stress-adaptive phenotype rather than a demonstrated anti-ferroptotic mechanism in bone.

Antioxidant signaling dependent on Nrf2 is one potential point of convergence because it regulates cellular redox defense and ferroptosis-related genes [7,89]. However, the available evidence does not establish an ERK5–Nrf2–GPX4 signaling axis in bone cells, and direct regulation of SLC7A11 or GPX4 by ERK5 has not been demonstrated in the skeletal system.

**Figure 2:**
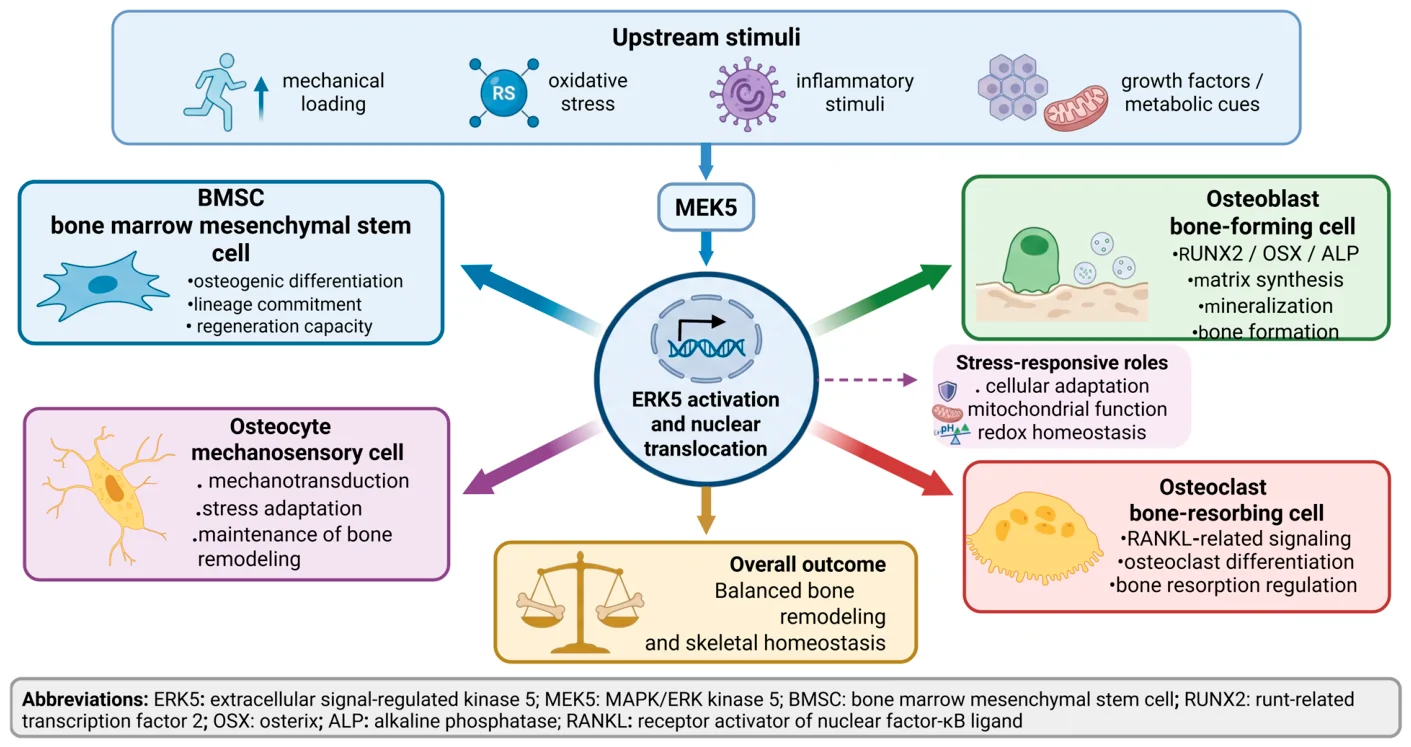
Biological functions of ERK5 signaling in skeletal homeostasis. Signaling through ERK5 integrates upstream stimuli, including mechanical loading, oxidative stress, inflammatory signals, and growth or metabolic cues, and regulates the functions of BMSCs, osteoblasts, osteocytes, and osteoclasts. Through effects on differentiation, mechanoadaptation, survival, redox balance, and remodeling, ERK5 contributes to skeletal homeostasis; however, the direction and magnitude of these effects depend on cell type and experimental context. Solid arrows indicate experimentally supported relationships, whereas dashed arrows indicate proposed or incompletely validated connections. Created in BioRender. Zhang, D. (2026) https://BioRender.com/6vykmrr. Agreement number: TP2AAKTQKC.

Importantly, direct ERK5-ferroptosis evidence has now emerged outside bone. A 2026 study demonstrated that ERK5 can promote microglial ferroptosis through an NFATc4-CLPTM1L mechanism in ischemic white-matter injury [25], whereas a separate study found that the apparent anti-ferroptotic effects of the ERK5 inhibitors XMD8-92 and JWG-045 were ERK5-independent [24]. These findings strengthen the rationale for investigating ERK5 in ferroptosis while simultaneously cautioning against extrapolating a single directional effect to skeletal cells. The major context-dependent functions of ERK5 in skeletal homeostasis are summarized in Figure 2.

## 4. Interactions Between ERK5 Signaling and Ferroptosis: A Context-Dependent Regulatory Framework

### 4.1. Stress-Responsive ERK5 Signaling and Ferroptotic Susceptibility

Ferroptosis is defined by iron-dependent phospholipid peroxidation rather than by ROS elevation alone [5,6,31]. Mitochondrial metabolism can modulate ferroptotic susceptibility under conditions in which oxidative phosphorylation, mitochondrial ROS production, or mitochondrial lipid metabolism substantially contribute to cellular redox balance. However, the requirement for mitochondria varies with the ferroptosis inducer, metabolic state, and cell type; for example, some cystine-deprivation-associated ferroptotic responses are strongly influenced by mitochondrial metabolism, whereas other ferroptotic programs can proceed largely independently of mitochondrial activity [90].

The kinase ERK5 (MAPK7/BMK1) is activated by growth factors, mechanical cues, and oxidative stress and contains a C-terminal transcriptional activation domain that permits kinase-dependent and transcription-associated responses [15,16,72,73]. These properties make ERK5 a plausible regulator of cellular state rather than a dedicated ferroptosis effector.

Evidence from stress-response studies links MEK5/ERK5 signaling to cellular adaptation, antioxidant responses, and survival [73,87,88]. Ferroptosis-specific interpretation requires separating ERK5-dependent mechanisms from ERK5-independent drug effects, as discussed in Section 4.3 [24,25].

### 4.2. Stress-Adaptive ERK5 Signaling and Resistance to Ferroptotic Stress

Moderate mechanical or oxidative stress may engage ERK5-associated adaptive programs [72,73,81,87,88]. Whether this adaptation changes the ferroptotic threshold in skeletal progenitors or osteoblast-lineage cells remains to be tested. Table 2 compares conflicting skeletal and non-skeletal findings relevant to this question [20,21,24,76].

**Table 2:** Context-dependent and apparently conflicting roles of ERK5 signaling relevant to bone homeostasis and ferroptotic susceptibility.

Study	Experimental Model	ERK5/MEK5 Manipulation and Dose/Exposure	Main Finding	Interpretation for the ERK5–Ferroptosis Hypothesis
Zhao et al., 2014 [19]	MC3T3-E1 osteoblasts under intermittent fluid shear stress	Mechanical stimulation; BIX02189-mediated MEK5 inhibition. FSS: 12 dyn/cm^2^.	Fluid shear stress activated MEK5/ERK5 and promoted osteogenic differentiation; pathway inhibition attenuated this response.	Supports mechanically induced MEK5–ERK5 pathway adaptation. Because BIX02189 can inhibit both MEK5 and ERK5, pharmacology alone establishes neither an ERK5-specific nor a ferroptosis-specific mechanism [91,92].
Kaneshiro et al., 2015 [76]	MC3T3-E1 and ST2 osteoblastic cells	BIX02189; MEK5 knockdown/overexpression. BIX02189 dose–response; proliferation decreased at concentrations >0.5 µM.	MEK5 inhibition or knockdown enhanced osteogenic differentiation and mineralization, whereas MEK5 overexpression suppressed differentiation.	Contrasts with mechanically stimulated osteoblast studies and indicates that MEK5/ERK5 output depends strongly on culture context and differentiation state.
Horie et al., 2022 [21]	Adult LepR^+^ BM-MSC-specific *Erk5* knockout mice and primary BM-MSCs	Conditional *Erk5* deletion; genetic model.	*Erk5* loss increased RUNX2/OSX expression, osteogenic differentiation, and bone formation, producing progressive osteosclerosis.	Strong genetic evidence that adult stromal ERK5 can restrain osteogenesis; highlights lineage- and stage-dependent ERK5 function rather than a uniformly anabolic role.
Li et al., 2025 [20]	Prx1-Cre; *Mapk7* flox/flox mice and mesenchymal stem cells	Genetic *Mapk7* deletion and overexpression.	*Mapk7* loss caused low bone mass and marrow adiposity; *Mapk7* promoted osteogenesis and suppressed adipogenesis through LRP6/β-catenin signaling.	Directly contrasts with adult LepR^+^ BM-MSC deletion and supports developmental- and lineage-specific ERK5 effects relevant to the proposed context-dependent model.
Amano et al., 2015 [22]	4B12, RAW264.7D, and M-CSF-dependent bone marrow macrophage models	BIX02189: ~4–5 µM; XMD8-92: 1 µM; MEK5 knockdown.	Pathway inhibition or MEK5 knockdown reduced c-Fos induction and osteoclast differentiation.	Supports a pro-osteoclastogenic ERK5 pathway but relies partly on pharmacology; micromolar inhibitor exposure and XMD8-92 off-target activity complicate ERK5-specific interpretation.
Loveridge et al., 2017 [23]	Nkx3.1:Cre-driven *Erk5*-deficient mice and primary marrow cultures	Partial genetic *Erk5* loss; BIX02188/BIX02189: 100 nM.	Reduced *Erk5* expression or pathway activity increased RANKL-induced osteoclastogenesis; mice showed increased resorption and trabecular osteopenia.	Opposes Amano et al.; differences in model, Cre specificity, and inhibitor concentration provide plausible explanations for the conflicting osteoclast phenotypes.
Zhang et al., 2026 [24]	Human cancer-cell datasets; breast cancer and HeLa cells with ERK5 silencing/knockout	XMD8-92: 5 µM; JWG-045: 3 µM; JWG-071: 3 µM; BAY-885: 5 µM; Fer-1: 1 µM; RSL3: 250 nM.	XMD8-92 and JWG-045 suppressed RSL3-induced ferroptosis even after ERK5 depletion.	Demonstrates that the anti-ferroptotic effects of these compounds can be ERK5-independent; inhibitor-based protection must not be interpreted as evidence that ERK5 itself suppresses ferroptosis.
Chu et al., 2026 [25]	Microglia in ischemic/demyelinating white-matter injury models	Genetic *Erk5*/Nfatc4/Clptm1l pathway perturbation; pharmacological ERK5 inhibition as complementary validation.	ERK5 promoted microglial ferroptosis through NFATc4 phosphorylation and CLPTM1L induction, with corresponding ferroptosis-related changes.	Provides direct mechanistic evidence that ERK5 can promote ferroptosis in a non-skeletal cell type, supporting a testable—but not established—pro-ferroptotic hypothesis in bone.

Interpretation: Kinase inhibition, genetic ERK5 depletion, and domain-specific perturbation are not interchangeable. The compound XMD8-92 has bromodomain activity; several direct ERK5 inhibitors can paradoxically activate the C-terminal TAD. The compounds BIX02188/BIX02189 were developed as MEK5 inhibitors but also inhibit ERK5 in biochemical assays [91,92], so they should be interpreted as pathway probes rather than strictly upstream or ERK5-specific interventions. Compound identity, exposure, lineage, loading conditions, and animal sex should therefore be reported when comparing skeletal studies. Abbreviations: ALP, alkaline phosphatase; BM-MSC, bone marrow mesenchymal stem cell; ERK5, extracellular signal-regulated kinase 5; FSS, fluid shear stress; GPX4, glutathione peroxidase 4; MEK5, mitogen-activated protein kinase kinase 5; OCN, osteocalcin; OPN, osteopontin; OSX, osterix; ROS, reactive oxygen species; RUNX2, runt-related transcription factor 2.

### 4.3. Context-Dependent Pro-Ferroptotic Effects Associated with ERK5 Signaling

Two non-skeletal studies clarify why ERK5-dependent biology must be separated from drug effects. The compounds XMD8-92 and JWG-045 are ATP-competitive small-molecule inhibitors developed to target ERK5, whereas RSL3 is a covalent inhibitor of glutathione peroxidase 4 (GPX4), the enzyme that detoxifies phospholipid hydroperoxides. In cancer cells, XMD8-92 and JWG-045 reduced RSL3-induced ferroptosis even after ERK5 had been depleted, showing that this protection was ERK5-independent rather than evidence that ERK5 itself suppresses ferroptosis [24].

By contrast, a neurological study identified a direct ERK5-dependent pro-ferroptotic mechanism: ERK5 promoted microglial ferroptosis by phosphorylating nuclear factor of activated T cells 4 (NFATc4) and inducing cleft lip and palate transmembrane protein 1-like (CLPTM1L) [25]. Thus, ERK5 can regulate ferroptosis in at least some non-skeletal cells, but neither study establishes the same mechanism in bone. The cancer-cell data primarily warn against equating ERK5-directed drug effects with ERK5 biology, whereas the microglial pathway provides a mechanistic hypothesis that remains to be tested in skeletal cells.

### 4.4. Mitochondrial Homeostasis and Ferroptotic Susceptibility Associated with ERK5

Mitochondria influence ferroptotic susceptibility through bioenergetic state, mitochondrial reactive oxygen species (ROS) generation, lipid metabolism, and organelle-specific antioxidant defense. Mitochondrial dysfunction should not be regarded as synonymous with ferroptosis [90,93].

Importantly, mitochondria possess ferroptosis-defense mechanisms that are partly distinct from those operating in other cellular compartments. Dihydroorotate dehydrogenase (DHODH), located in the inner mitochondrial membrane, reduces ubiquinone (CoQ) to ubiquinol and suppresses mitochondrial lipid peroxidation. The mitochondrial enzyme DHODH acts in parallel with mitochondrial GPX4, whereas FSP1 predominantly provides an extra-mitochondrial CoQ-dependent defense system [37,93]. These findings indicate that ferroptotic susceptibility can depend not only on total cellular antioxidant capacity but also on the subcellular distribution of GPX4- and CoQ-dependent defense systems.

The ERK5 pathway provides a plausible upstream interface with these mitochondrial processes, but the available evidence remains indirect. Mitochondrial complex I activity has been reported to engage ERK5-dependent antioxidant signaling [73], whereas MEKK3–MEK5–ERK5 signaling has also been linked to mitochondrial degradation [94]. These observations suggest that ERK5 may alter mitochondrial state through more than one mechanism, but neither study established a direct effect on ferroptotic execution.

Complex I can contribute to both adaptive signaling [73] and mitochondrial ROS generation, particularly during reverse electron transport [95]. The balance therefore depends on substrate availability, membrane potential, and metabolic state.

In osteoblasts, mitochondrial ferritin deficiency has been linked to enhanced mitophagy, iron dysregulation, and ferroptotic injury in experimental diabetic osteoporosis [96]. This provides a skeletal rationale for testing whether the MEKK3–MEK5–ERK5 mitochondrial-degradation pathway affects ferroptotic susceptibility. Its effects on mitophagy, mitochondrial iron handling, and mitochondrial ferroptosis defenses remain to be established in skeletal cells.

A compartment-specific test would compare responses to mitochondrial GPX4/DHODH impairment and predominantly extra-mitochondrial FSP1 impairment after ERK5 gain- or loss-of-function. ERK5 activity, mitochondrial respiration and ROS, DHODH/CoQ status, GPX4 activity, lipid peroxidation, and ferroptosis-specific rescue should be assessed together [37,93].

### 4.5. A Proposed Context-Dependent Model of ERK5-Mediated Ferroptosis Regulation in Bone Homeostasis

**Figure 3:**
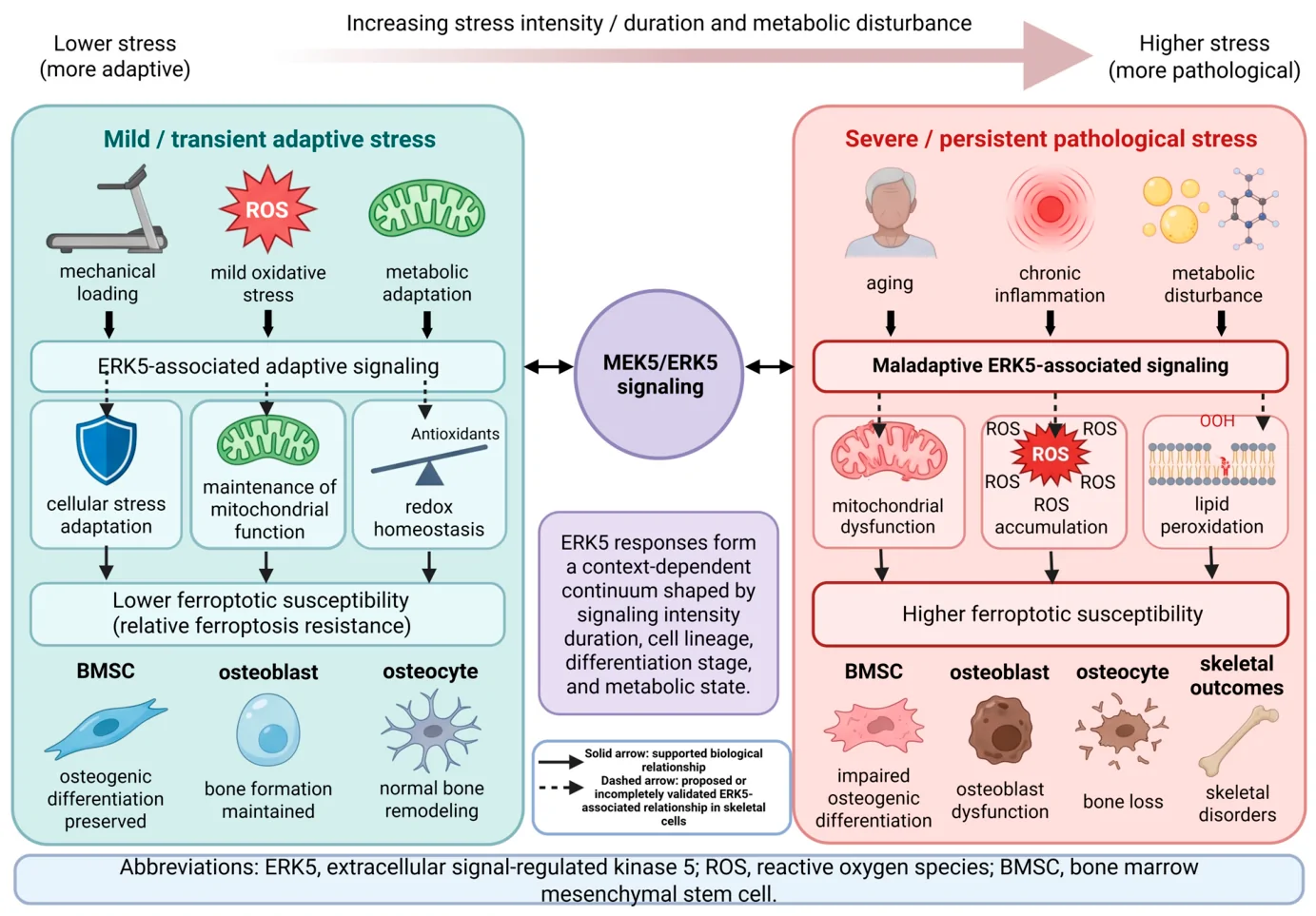
Context-dependent continuum of ERK5-associated ferroptotic susceptibility during bone remodeling. Mild or transient stress and severe or persistent stress are presented as opposite ends of a graded continuum of proposed ERK5-associated responses, shaped by stress intensity and duration, cell lineage, differentiation stage, and metabolic state. “Maladaptive ERK5-associated signaling” refers to alterations in signaling intensity, duration, subcellular localization, or the balance between kinase and C-terminal transcriptional outputs, rather than a uniformly increased or decreased ERK5 state. The solid arrows from severe or persistent pathological stress (aging, chronic inflammation, and metabolic disturbance) to “maladaptive ERK5-associated signaling” represent hypothetical relationships in skeletal cells; their solid appearance does not indicate experimental validation. Dashed arrows denote other proposed or incompletely validated ERK5-associated relationships. Other solid arrows summarize supporting biological relationships and do not establish a complete ERK5-dependent ferroptosis pathway in bone. Direct causal links between ERK5-associated adaptive or maladaptive signaling and ferroptotic susceptibility remain unproven in skeletal cells. Created in BioRender. Zhang, J. (2026). https://BioRender.com/vpeo9sr. Agreement number: AQ2AAKTGX2.

Figure 3 presents a proposed continuum: mild or transient stress may favor adaptive ERK5-associated responses, whereas severe or persistent stress may accompany maladaptive signaling and greater ferroptotic susceptibility [25,73,81,87,88]. The latter branch is hypothetical in skeletal cells. The model motivates lineage-specific tests rather than assigning ERK5 a uniformly protective or harmful role [24,25].

BMSC lineage allocation offers a specific point of convergence. In senescent BMSCs, *Steap4*-dependent Fe^2+^/ROS signaling has been reported to couple ferroptotic stress with enhanced adipogenic differentiation [43]. Independently, mesenchymal *Mapk7* deletion caused low bone mass and increased marrow adiposity, whereas *Mapk7* promoted osteogenesis through LRP6/β-catenin signaling [20]. These observations concern separate pathways but converge on the osteogenic-to-adipogenic shift. They motivate testing whether *Mapk7* gain- or loss-of-function alters *Steap4* expression, intracellular Fe^2+^, lipid peroxidation, and lineage allocation in senescent or metabolically stressed BMSCs, together with ferroptosis-selective rescue. They do not establish a direct ERK5–*Steap4*–ferroptosis mechanism.

## 5. Skeletal Disorders: Disease-Relevant Implications of ERK5 and Ferroptosis

### 5.1. Osteoporosis: Intersections Between ERK5 Signaling and Ferroptosis

Osteoporosis is accompanied by an imbalance between bone formation and resorption, and many preclinical models also show oxidative stress, disturbed iron handling, and ferroptosis-related injury in osteoblasts, osteocytes, and BMSCs [14,40,43,47,58,97]. Aging, metabolic disease, and estrogen deficiency can therefore create conditions that favor lipid peroxidation and impaired antioxidant defense.

Estrogen deficiency increases osteoclast-mediated resorption and can be accompanied by oxidative and inflammatory stress. It therefore provides a setting for testing how ERK5 perturbation affects ferroptotic susceptibility, rather than evidence that estrogen directly regulates ERK5 [19,20,21,76,79].

Clinical iron-overload disorders provide supporting context but not direct proof of ferroptosis. Hereditary hemochromatosis and transfusion-dependent β-thalassemia are both associated with low bone mass and fracture risk in settings of chronic iron excess [98,99], and iron chelation is part of systemic management in thalassemia [100]. These observations reinforce the relevance of iron dysregulation to skeletal deterioration without establishing ferroptosis as the causal mechanism in patients.

### 5.2. Age-Related Skeletal Degeneration: ERK5 and Ferroptosis

Skeletal aging combines mitochondrial dysfunction, oxidative stress, senescence, altered iron handling, and declining progenitor function. Ferroptosis-related mechanisms have been reported in aged osteocytes and BMSCs, including ATF3/TFR1-driven iron uptake, *Steap4*-dependent Fe^2+^/ROS signaling, and DPP4-associated ferroptotic senescence [43,52,63].

Senescence, however, should not be equated automatically with greater ferroptotic sensitivity. Senescent cells can persist despite chronic stress, while age-associated changes in iron metabolism, lipid handling, mitochondria, and antioxidant capacity could either increase or decrease the ferroptotic threshold. The available studies therefore support mechanistic coupling between senescence and ferroptosis but do not establish a uniform direction of susceptibility [43,63].

For the ERK5 hypothesis, senescent and non-senescent skeletal cells should be compared under matched ferroptotic challenge, with ERK5 gain- and loss-of-function tested in both states.

### 5.3. Fracture Healing and Bone Regeneration: ERK5-Ferroptosis Interactions

Fracture repair requires coordinated inflammation, progenitor recruitment, angiogenesis, osteogenic differentiation, and remodeling [101,102]. Controlled redox signaling participates in repair, whereas excessive oxidative stress and ferroptotic injury can impair osteoblast or progenitor function and delay healing in experimental fracture or nonunion models [64,65].

A phase-resolved hypothesis yields four distinct tests. During the inflammatory phase, ERK5-associated stress adaptation and endothelial responses may become important under inflammatory or hypoxic stress; prolonged stress is predicted to increase ferroptotic injury in recruited progenitors or vascular cells. During soft-callus formation, the developmental Smurf–Smad–Sox9 evidence motivates testing ERK5 in progenitor expansion and chondrogenic differentiation [79], while ferroptotic injury is predicted to reduce the viable progenitor pool and callus formation. During hard-callus formation, osteoblast differentiation, vascular invasion, and mechanotransduction become central [19,72,81,82]; ferroptotic osteoblast or endothelial injury is predicted to impair matrix deposition, mineralization, or vascularized bone formation. During remodeling, coordinated osteoblast–osteoclast activity is required [22,80,101], and ferroptosis suppression may preserve osteoblast function while also prolonging osteoclast survival and resorption. These are stage-specific predictions, not demonstrated ERK5–ferroptosis pathways; they require longitudinal, lineage-resolved testing.

Bone endothelium is another plausible compartment. The kinase ERK5 supports endothelial survival and flow-responsive KLF2/KLF4 signaling [103,104,105], while CD31-high/Emcn-high type H vessels couple angiogenesis to osteogenesis in bone [106]. Because endothelial ferroptosis occurs in non-skeletal vascular disease [107], testing whether ERK5 modifies ferroptotic susceptibility in type H endothelium could clarify whether vascular stress contributes to impaired fracture repair.

Longitudinal ERK5 and ferroptosis-specific measurements, bone-cell- or endothelial-specific ERK5 perturbation, and ferroptosis-selective rescue should be aligned with these healing phases.

### 5.4. Inflammatory Bone Disorders: ERK5 and Ferroptosis

Inflammatory bone loss involves cytokine-driven changes in redox balance, iron handling, and osteoblast–osteoclast communication [108,109,110]. The kinase ERK5 can support anti-inflammatory transcriptional programs involving KLF2/KLF4 and PPARδ in non-skeletal systems [104,105,111], making inflammatory stress a plausible setting in which ERK5 state could modify ferroptotic susceptibility.

The contribution of ferroptosis should be assessed separately in each inflammatory bone-disease model. Cytokine-driven effects on bone-cell function should be distinguished from ERK5-dependent changes in ferroptotic susceptibility.

## 6. Therapeutic Perspectives and Future Directions

### 6.1. Targeting Ferroptosis for Skeletal Disease Intervention

Ferroptosis provides a tractable experimental framework for skeletal disorders characterized by iron and redox imbalance [26,112]. Across BMSCs, osteoblasts, osteocytes, and osteoclast precursors, preclinical studies identify lipid peroxidation and impaired antioxidant defense as modifiable contributors to cellular dysfunction [10,13,14,58].

Therapeutic approaches tested experimentally include iron chelation, lipid-radical trapping with ferrostatin-1 or liproxstatin-1, and restoration of endogenous antioxidant defenses [11,14,40,49,113]. These interventions can improve osteogenic or skeletal outcomes in selected models [14,40,49,64], but their efficacy has not been established clinically.

Melatonin, an endogenous indoleamine with antioxidant and circadian functions, is particularly relevant because preclinical skeletal studies have directly linked it to ferroptosis modulation. In high-glucose osteoblast and diabetic osteoporosis models, melatonin reduced lipid peroxidation and restored osteogenic capacity through Nrf2/HO-1 signaling [114]. In glucocorticoid-exposed BMSCs and a steroid-induced osteoporosis rat model, melatonin reduced ferroptosis through PI3K/AKT/mTOR signaling and improved skeletal outcomes [115]. These findings identify melatonin as a clinically familiar and generally well-tolerated candidate for ferroptosis-directed intervention [116], but the evidence remains preclinical, and effective bone-specific dosing, long-term efficacy, and effects on osteoclasts require further study.

These findings do not justify systemic ferroptosis suppression as a default strategy. Ferroptosis is embedded in normal iron, lipid, and redox physiology, and its consequences differ across skeletal cell types [5,59]. In osteoblasts and osteocytes, limiting excessive ferroptosis may preserve bone formation, whereas in osteoclasts the same intervention could prolong survival or differentiation and increase resorption [11,13,14,49,54,55,57,58].

Accordingly, translational studies should assess both formation and resorption and should favor cell- and stage-specific intervention. Bone-formation indices, osteoblast/osteocyte survival, osteoclast number and resorptive activity, and overall remodeling balance should be measured together before any systemic ferroptosis-directed therapy is considered.

### 6.2. Therapeutic Potential and Challenges of Targeting ERK5 Signaling

The kinase ERK5 is an attractive but difficult therapeutic target because it regulates differentiation, stress responses, and tissue remodeling while producing divergent skeletal phenotypes across experimental systems [18,20,21,22,23].

Pharmacological interpretation depends on which ERK5 function and additional targets a compound affects. The inhibitor XMD8-92 binds bromodomain proteins, including BRD4 [117]. More kinase-selective compounds can still promote ERK5 nuclear localization and paradoxical TAD activation while suppressing catalytic activity [74,75]. The compounds BIX02188/BIX02189 were developed as MEK5 inhibitors but also inhibit ERK5 in biochemical assays [91,92]. Although BIX02189 did not induce paradoxical TAD activation in the Lochhead experimental system [74], this does not imply an absence of direct ERK5 binding or establish selectivity in skeletal cells. Table 3 summarizes the compound-specific evidence. No ERK5-directed treatment is currently validated for skeletal disease.

**Table 3:** Representative pharmacological tools targeting the MEK5–ERK5 pathway and key interpretive limitations.

Compound	Nominal Target/Class	Representative Potency	Documented Off-Targets/ Key Limitations	Paradoxical ERK5 TAD Activation	Evidence in Skeletal Systems
BIX02188	MEK5–ERK5 pathway inhibitor	MEK5 IC50: 4.3 nM; ERK5 IC50: 810 nM (biochemical assays) [91,92]	Also inhibits ERK5 and additional kinases; dose and assay context limit target-specific interpretation [91,92].	Not characterized in the cited paradoxical-activation assay; absence of evidence is not evidence of absence.	Used in osteoclast studies, including Loveridge et al. [23].
BIX02189	MEK5–ERK5 pathway inhibitor	MEK5 IC50: 1.5 nM; ERK5 IC50: 59 nM (biochemical assays) [91,92]	Direct ERK5 activity prevents a strictly upstream interpretation; other kinase effects depend on concentration [91,92].	No paradoxical TAD activation observed in the Lochhead et al. system [74].	Used in osteoblast and osteoclast studies, including Zhao, Kaneshiro, Amano, and Loveridge [19,22,23,76].
XMD8-92	ATP-competitive ERK5 inhibitor; dual ERK5/BRD pharmacology	ERK5 IC50: ~190 nM [75,117]	BRD4 binding is comparable to ERK5 inhibition (BRD4 Kd ≈ 170 nM); LRRK2 and other kinase off-targets are also reported [75,117].	Reported in the ERK5–MEF2D reporter assay; magnitude is assay dependent [75].	Used in osteoclast/osteoblast studies [22,76]; anti-ferroptotic activity can persist after ERK5 depletion in non-skeletal cells [24].
XMD17-26 (compound 25)	ATP-competitive ERK5 inhibitor with BRD4 activity	ERK5 IC50: ~82 nM in vitro [118]	Documented BRD4 activity confounds ERK5-specific interpretation; kinase inhibition is distinct from protein loss [75,119].	Reported [74].	No direct skeletal application identified in the reviewed literature.
XMD17-109 (compound 26; ERK5-IN-1)	ATP-competitive ERK5 inhibitor with dual ERK5/BRD activity	ERK5 IC50: ~162 nM [118]	BRD4 binding (~217 nM) and LRRK2 inhibition (~339 nM) complicate ERK5-specific interpretation [75,118].	Reported [74].	No direct skeletal application identified in the reviewed literature.
AX15836	More selective ATP-competitive ERK5 inhibitor	ERK5 IC50: ~8 nM [117]	Markedly reduced BRD4 activity (BRD4 Kd ≈3.6 μM) compared with XMD8-92, but selective kinase inhibition still does not reproduce ERK5 protein loss [75,117].	Reported in the Lochhead system [74].	No direct skeletal application identified in the reviewed literature.
JWG-045 (XMD10-78)	ATP-competitive ERK5 inhibitor	ERK5 IC50: ~98 nM [75,119]	Improved ERK5/BRD4 separation, but residual LRRK2 and other off-target activity remains; anti-ferroptotic effects can be ERK5-independent [24].	Not characterized in the cited TAD assays.	No direct skeletal validation; used in non-skeletal ferroptosis studies [24].
JWG-071	ERK5-focused ATP-competitive inhibitor	ERK5 IC50: ~88 nM [119]	Improved BRD4 selectivity, but residual LRRK2 and DCLK-family activity has been reported [119].	Not characterized in the cited TAD assays.	No direct skeletal validation; used as a comparator in non-skeletal ferroptosis studies [24].
BAY-885	Potent/selective ATP-site ERK5 inhibitor	ERK5 IC50: ~35 nM; cellular MEF2 reporter IC50: ~120 nM [120]	Minimal BRD4 activity relative to earlier probes [120], but paradoxical TAD activation remains a limitation [75].	Reported in the ERK5–MEF2D reporter assay (Cook et al., Figure 4A) [75].	No direct skeletal validation; used as a comparator in non-skeletal ferroptosis studies [24].

Note: Potency values originate from biochemical, chemoproteomic, and cellular assays and are not directly interchangeable. Reported paradoxical TAD activation refers to the specified non-skeletal experimental system; it has not been established in bone cells for every listed compound. “Not characterized” means that the cited studies do not establish the response, not that the response is absent. TAD, transcriptional activation domain; IC50, half-maximal inhibitory concentration; Kd, dissociation constant.

**Figure 4:**
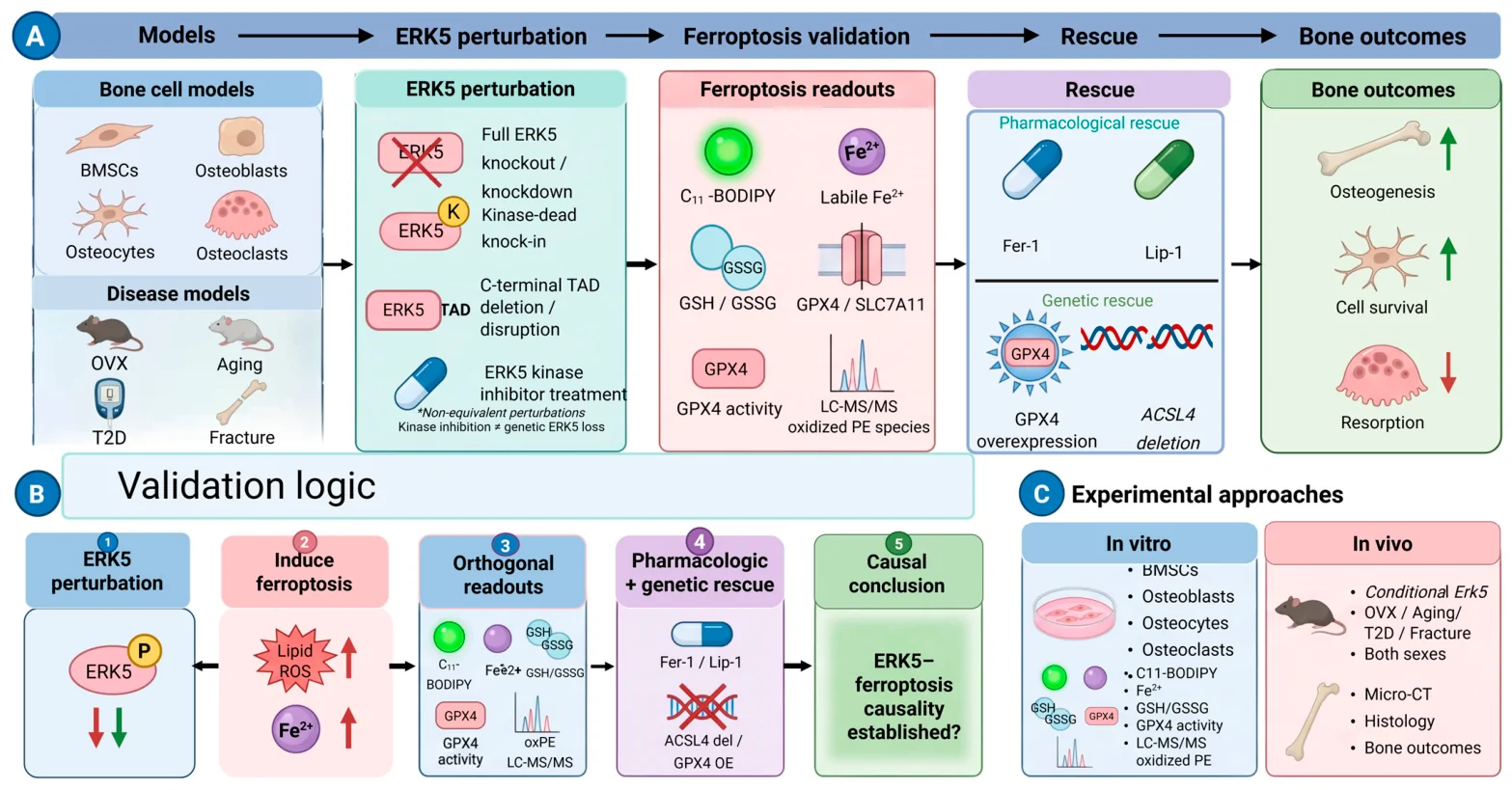
Experimental framework for validating ERK5–ferroptosis interactions in skeletal cells. (**A**) Proposed workflow using BMSCs, osteoblasts, osteocytes, and osteoclasts under physiological or disease-related conditions. Four non-equivalent ERK5 perturbations are shown: full knockout or knockdown, kinase-dead knock-in, TAD deletion or disruption, and inhibitor treatment. Ferroptosis-related measurements are combined with pharmacological rescue using ferrostatin-1 (Fer-1) or liproxstatin-1 (Lip-1), genetic rescue using GPX4 overexpression or *Acsl4* deletion, and assessment of skeletal functional outcomes. (**B**) Recommended validation logic combining ERK5 perturbation, ferroptosis induction, orthogonal readouts, rescue experiments, and functional phenotyping to test causality rather than marker association alone. (**C**) Representative in vitro and in vivo approaches include lipid ROS, labile Fe^2+^, GSH/GSSG, GPX4 and SLC7A11 measurements, GPX4 activity, oxidized phospholipid analysis, and conditional *Erk5* models with micro-CT and histological skeletal phenotyping. The illustrated bone-outcome directions are proposed therapeutic goals, not established effects of ERK5 modulation or systemic ferroptosis suppression. Additional requirements for causal validation are detailed in Section 6.4. Horizontal arrows indicate the proposed experimental workflow or sequence of validation steps. Circled P denotes phosphorylation, whereas circled K identifies the kinase-dead ERK5 construct. Abbreviations: BMSCs, bone marrow mesenchymal stem cells; ERK5, extracellular signal-regulated kinase 5; TAD, transcriptional activation domain; Fer-1, ferrostatin-1; Lip-1, liproxstatin-1; GPX4, glutathione peroxidase 4; SLC7A11, solute carrier family 7 member 11; GSH, glutathione; GSSG, oxidized glutathione; ROS, reactive oxygen species; OVX, ovariectomy; T2D, type 2 diabetes. Created in BioRender. Zhang, D. (2026) https://BioRender.com/wn7o1wy. Agreement number: HY2AAKTZ5O.

The target cell population, disease stage, and affected ERK5 function must therefore be specified before combining pathway-directed compounds with ferroptosis-modifying interventions [20,21,74,75,117].

### 6.3. Therapeutic Integration of ERK5 Modulation and Ferroptosis Regulation

Combined ERK5 modulation and ferroptosis-directed intervention remains a therapeutic hypothesis [24,25]. Evaluation should distinguish ERK5-dependent effects from drug-specific actions and measure both cell survival and skeletal function.

### 6.4. Experimental Approaches to Validate ERK5–Ferroptosis Interactions

Direct validation should treat ERK5 protein abundance, kinase activity, and C-terminal transcriptional activity as separable variables [74,75]. Ideally, experiments should compare full ERK5 knockout or knockdown, kinase-dead knock-in, C-terminal TAD deletion or disruption, and pharmacological inhibitor treatment rather than assuming that these perturbations are functionally equivalent [74,75]. In vivo work should prioritize bone-cell-specific conditional *Erk5* models in ovariectomy, aging, metabolic bone disease, and fracture-healing settings [20,21]. Animal sex should be reported explicitly, and both sexes should be evaluated where biologically and experimentally appropriate to determine whether ERK5-dependent ferroptotic susceptibility is sex dependent.

Mitochondrial profiling should accompany ERK5 perturbation because respiratory state is a candidate modifier of the response. Measurements should include complex I activity, mitochondrial respiration, and, where feasible, complex I subunit or assembly-factor expression. Skeletal lineages and differentiation states should be compared directly.

Ferroptosis validation should combine direct cell-death assessment with orthogonal readouts. Cell-death measurements may include membrane-integrity or viability assays with quantitative death assessment. Biochemical readouts should include C11-BODIPY lipid peroxidation, labile Fe^2+^, GSH/GSSG, GPX4/SLC7A11 expression, and GPX4 activity; GPX4 abundance alone does not indicate its functional capacity [59]. Where feasible, LC-MS/MS should quantify oxidized arachidonoyl- and adrenoyl-containing phosphatidylethanolamines [31]. Pharmacological rescue with ferrostatin-1 or liproxstatin-1 [113] should be paired with genetic approaches, such as *Acsl4* deletion or GPX4 overexpression [6,30], and parallel assessment of apoptosis, necroptosis, or other cell injury [29,59]. Concordant cell-death, biochemical, lipidomic, and rescue results provide stronger support for ferroptotic causality than any single marker or rescue strategy. In ERK5 loss-of-function experiments, wild-type ERK5 re-expression should test specificity, with kinase-dead or transcriptionally altered constructs used to resolve the contributing ERK5 function [74,75].

Before ferroptotic challenge, ERK5-manipulated and control cells should be compared for baseline cell number, viability, proliferation, and differentiation. Challenge conditions and rescue analyses should be matched or normalized to these differences so that changes in cellular state are not misattributed to altered ferroptotic susceptibility.

In osteocytes, the ERK5–MEF2C–SOST relationship provides a more specific mechanistic test. Perturbation of ERK5 should be combined with assessment of MEF2C activation and nuclear localization, MEF2C occupancy at the ECR5 enhancer by ChIP-qPCR, *Sost/SOST* expression, sclerostin secretion, and downstream Wnt signaling. These measurements should then be paired with ferroptosis-specific phenotyping and rescue experiments to determine whether ERK5-dependent changes in the MEF2C–SOST transcriptional program are associated with, or mechanistically separable from, changes in osteocyte ferroptotic susceptibility [83,84].

Bone endothelial cells should also be incorporated into future validation studies. Endothelial-specific *Erk5* manipulation could be combined with assessment of CD31^hi^Emcn^hi^ type H vessels, vascular density and perfusion, endothelial lipid peroxidation, GPX4 activity, and ferroptosis-selective rescue. In fracture models, these measurements should be integrated with osteoprogenitor abundance, callus vascularization, and bone-formation outcomes to determine whether an endothelial ERK5–ferroptosis mechanism contributes to angiogenesis–osteogenesis coupling [106].

### 6.5. Testable Predictions and Outstanding Questions

The proposed framework generates several experimentally testable predictions.

Prediction 1: ERK5-dependent stress adaptation should modify the threshold for ferroptotic injury rather than uniformly suppress ferroptosis. Under mild or transient oxidative or mechanical stress, ERK5 loss is predicted to lower the threshold for ferroptotic injury in selected osteogenic cell populations, whereas restoration of ERK5 should partially restore stress tolerance. This effect should be demonstrated using ferroptosis-selective readouts and rescue rather than ROS or GPX4 expression alone. Comparison of wild-type ERK5 with kinase-dead or C-terminal transcriptionally altered constructs would further determine which ERK5 function is required.

Prediction 2: The direction and magnitude of ERK5-dependent ferroptotic susceptibility should vary across skeletal cell lineages and differentiation states. The same ERK5 perturbation is therefore predicted to produce different ferroptotic responses in BMSCs, osteoblasts, osteocytes, and osteoclast-lineage cells. Such differences should be tested under matched stress conditions and accompanied by lineage-specific functional outcomes, including osteogenesis, matrix formation, osteocyte integrity, and osteoclast-mediated resorption.

Prediction 3: Mitochondrial state should modify the ferroptotic consequences of ERK5 perturbation. If mitochondrial respiratory capacity is an important determinant of ERK5-dependent stress adaptation, then the magnitude or direction of ERK5-associated ferroptotic susceptibility should vary with complex I activity, respiratory capacity, and differentiation-dependent mitochondrial state. Manipulating mitochondrial function should therefore alter the ferroptotic phenotype produced by ERK5 gain- or loss-of-function.

## 7. Limitations and Methodological Considerations

The central evidentiary limitation is the absence of causal ERK5–ferroptosis data in skeletal cells. Non-skeletal findings [24,25] motivate the framework but do not establish it in bone.

The skeletal ERK5 literature relies mainly on murine systems, including MC3T3-E1, ST2, RAW264.7/RAW264.7D, and 4B12 cells and mouse genetic models [19,20,21,22,23,76,80]. Human bone marrow-derived skeletal precursors have been studied [77], but ERK5–ferroptosis interactions require validation in primary human skeletal cells and bone tissue. Differences in iron handling, redox metabolism, differentiation, and mitochondrial function may limit generalization from immortalized cell lines.

Future in vivo studies should clearly report the sex of the animals in accordance with the ARRIVE 2.0 guidelines [121]. Where scientifically appropriate, inclusion of both sexes and sex-stratified analyses should be considered.

The pharmacological limitations described in Section 6.2 require genetic and domain-specific confirmation, as outlined in Section 6.4 [74,75,117].

Translation remains limited by predominantly preclinical evidence [26,59]. The critical narrative design and descriptive evidence profiles provide a qualitative synthesis rather than systematic-review estimates, formal GRADE classifications, or quantitative quality scores.

## 8. Conclusions

Taken together, the current literature supports ferroptosis as a functionally important contributor to skeletal cell dysfunction in multiple preclinical models, while ERK5 is established as a regulator of skeletal development, mechanoadaptation, differentiation, and stress responses. What remains unproven is a direct causal ERK5–ferroptosis pathway in bone.

The next step is therefore not further associative comparison but causal testing. Bone-cell-specific ERK5 genetics, domain-resolved ERK5 perturbation, rigorous ferroptosis phenotyping, and temporally resolved disease models should determine whether ERK5 directly sets ferroptotic susceptibility or instead modifies broader cellular states that secondarily influence ferroptosis. This distinction will determine whether ERK5 is a therapeutically actionable ferroptosis regulator or primarily a context marker of skeletal stress.

## Figures and Tables

**Table 1:** Current evidence for ferroptosis in bone-related cells and skeletal disorders.

Cell Type	Disease/ Model	Ferroptotic Stimulus	Main Markers	Intervention	Major Finding	Evidence Profile and Representative References
Osteoblast	Iron-overload osteoporosis; diabetic bone disease; glucocorticoid exposure	Iron overload; high glucose/high fat; glucocorticoids; inflammatory stress	Fe^2+,^ ROS and lipid peroxidation ↑; GSH, SLC7A11 and GPX4 ↓; RUNX2, ALP and OCN ↓	Fer-1 or Lip-1; iron chelation; activation of Nrf2-related antioxidant defense	Osteoblast ferroptosis impairs survival, differentiation and mineralization and contributes to reduced bone formation; rescue of ferroptosis can improve osteogenic and skeletal outcomes.	Cellular lipid-peroxidation and rescue findings; selected studies also report skeletal outcomes. Osteoblast-specific genetic validation and primary human replication remain limited [11,45,46,47,48].
Osteoclast	RANKL-induced osteoclastogenesis; iron-overload models	RANKL stimulation; iron overload; ferritinophagy; pharmacological ferroptosis induction	ROS and lipid peroxidation; ferritinophagy; Nrf2/GPX4; altered TRAP, NFATc1 and cathepsin K	Hypoxia/ferritinophagy modulation; artesunate; saikosaponin A; Nrf2/GPX4 modulation	Ferroptosis generally limits osteoclast survival or differentiation, whereas iron loading and adaptive redox responses can simultaneously promote osteoclastogenesis, producing context-dependent net effects.	Cellular induction and intervention studies support involvement in osteoclast survival or differentiation. Lineage-specific genetic validation and ferroptosis-specific in vivo rescue remain limited; resorption effects depend on context [13,54,56,57].
Osteocyte	Diabetic, postmenopausal, age-related and iron-dysregulated bone loss	Glucolipotoxicity; estrogen withdrawal; ageing-related iron accumulation; LCN2-mediated iron loading	Intracellular iron/TFR1 ↑; lipid peroxidation ↑; GPX4/SLC7A11-related defense ↓; mitochondrial injury	Ferroptosis inhibition; GPX4-related genetic manipulation; ATF3 or LCN2 pathway modulation	Osteocyte ferroptosis disrupts cell viability and remodeling signals and contributes to trabecular or cortical deterioration and impaired bone formation.	Includes osteocyte-related GPX4 genetic perturbation and in vivo skeletal phenotyping [14], alongside diabetic, aging, and iron-loading mechanisms. Primary human osteocyte validation remains limited [12,14,52,53].
BMSC	Osteoporotic, iron-overloaded and senescent BMSCs; impaired BMP responsiveness	Iron overload; senescence; erastin; chronic oxidative/metabolic stress	Fe^2+,^ ROS and lipid peroxidation ↑; altered FTH1; SLC7A11/GSH/GPX4 defense ↓; osteogenic markers ↓	Fer-1; iron chelation; Nrf2-directed interventions; FTH1 stabilization; BMP9/GPX4 restoration	Ferroptotic injury reduces BMSC survival and osteogenic competence; senescent BMSCs can also couple ferroptosis to an adipogenic shift in a *Steap4*-dependent model.	Functional rescue of viability or osteogenesis is reported in several models. The CRYAB–FTH1 study uses iron/ROS and metabolic viability readouts with rescue [10]; direct death and lipid-peroxidation assessment varies across studies. BMSC-specific in vivo validation remains limited [10,39,40,43,63].
Osteoblasts, BMSCs and osteocytes	Osteoporosis	Iron overload; ageing; estrogen deficiency; abnormal iron storage	Iron, ROS and lipid peroxides ↑; Nrf2/SLC7A11/GPX4 defense ↓; osteogenic markers ↓	Ferroptosis inhibitors; iron chelators; activation of Nrf2/GPX4 defense	Ferroptosis in bone-forming and regulatory cells contributes to impaired bone formation and bone loss; inhibition improves skeletal outcomes across several preclinical osteoporosis models.	Includes in vivo skeletal outcomes with pharmacological or genetic intervention in selected models. These overlap with cell-type rows and do not establish clinical efficacy [14,39,43,58,66].
Osteoblasts, BMSCs and osteocytes	Diabetic bone disease	Hyperglycemia; high glucose/high fat; advanced glycation end products; inflammation	Labile iron, ROS and lipid peroxidation ↑; GSH, SLC7A11 and GPX4 ↓	Maresin 1; metformin; ferroptosis inhibitors; activation of Nrf2/GPX4 signaling	Ferroptotic injury impairs osteogenesis, mineralization and bone quality under diabetic conditions	Cellular mechanistic and intervention findings are accompanied by animal skeletal phenotyping in selected studies. Genetic replication across models and human skeletal validation remain limited [12,45,67,68].
Osteoblasts, osteocytes and BMSCs	Osteonecrosis	Glucocorticoids; dexamethasone; ischemia/hypoxia; microcirculatory injury	ROS and lipid peroxides ↑; altered p53/SLC7A11/GPX4 and Nrf2 signaling	Inhibition of lipid peroxidation; SLC7A11/GPX4 or Nrf2 modulation; miRNA-based interventions	Glucocorticoid-associated ferroptotic injury impairs osteogenesis and bone-cell survival and contributes to experimental osteonecrosis.	Intervention and pathway findings are concentrated in glucocorticoid-associated experimental models. Lineage-specific genetic causality and ferroptosis-directed clinical evidence remain limited [46,61,62].
Skeletal stem/progenitor cells and osteoblasts	Impaired fracture healing or nonunion	Excessive inflammation; IL-1β-associated stress; ischemia/hypoxia; oxidative stress	Lipid peroxidation ↑; intracellular Fe2+ ↑; ferroptosis-related proteins; impaired osteochondrogenic/osteoblastic function	Ferroptosis inhibition; Nrf2-related interventions; EPAS1 inhibition in nonunion models	Ferroptotic stress can impair osteoblast or skeletal progenitor function and delay repair, but the optimal target cell and therapeutic window remain uncertain.	Preclinical functional findings in fracture or nonunion models. Stage-specific in vivo rescue, independent replication, and clinical validation are still needed [64,65].

↑ and ↓ indicate increases and decreases, respectively, relative to the corresponding control or comparison condition. Abbreviations: ALP, alkaline phosphatase; BMSC, bone marrow mesenchymal stem cell; Fer-1, ferrostatin-1; FTH1, ferritin heavy chain 1; GPX4, glutathione peroxidase 4; GSH, glutathione; Lip-1, liproxstatin-1; NFATc1, nuclear factor of activated T cells 1; OCN, osteocalcin; ROS, reactive oxygen species; RUNX2, runt-related transcription factor 2; SLC7A11, solute carrier family 7 member 11; TFR1, transferrin receptor 1; TRAP, tartrate-resistant acid phosphatase.

## Data Availability

No new data were created or analyzed in this study. Data sharing is not applicable to this article.

## Abbreviations

ACSL4	acyl-CoA synthetase long-chain family member 4
ALP	alkaline phosphatase
BMSCs	bone marrow mesenchymal stem cells
ERK5	extracellular signal-regulated kinase 5
Fer-1	ferrostatin-1
GPX4	glutathione peroxidase 4
GSH	glutathione
HO-1	heme oxygenase 1
KLF2	Krüppel-like factor 2
KLF4	Krüppel-like factor 4
Lip-1	liproxstatin-1
MAPK7	mitogen-activated protein kinase 7
M-CSF	macrophage colony-stimulating factor
MEF2	myocyte enhancer factor 2
Nrf2	nuclear factor erythroid 2-related factor 2
OCN	osteocalcin
PUFA	polyunsaturated fatty acid
PUFA-PL	polyunsaturated fatty acid-containing phospholipid
RANKL	receptor activator of nuclear factor-κB ligand
ROS	reactive oxygen species
RUNX2	runt-related transcription factor 2
SLC7A11	solute carrier family 7 member 11
STEAP3	six-transmembrane epithelial antigen of prostate 3
*Steap4*	six-transmembrane epithelial antigen of prostate 4
system Xc^−^	cystine/glutamate antiporter
TFR1	transferrin receptor 1
TRAP	tartrate-resistant acid phosphatase
